# The Effects of Sampling Bias and Model Complexity on the Predictive Performance of MaxEnt Species Distribution Models

**DOI:** 10.1371/journal.pone.0055158

**Published:** 2013-02-14

**Authors:** Mindy M. Syfert, Matthew J. Smith, David A. Coomes

**Affiliations:** 1 Forest Ecology and Conservation Group, Department of Plant Sciences, University of Cambridge, Cambridge, United Kingdom; 2 Computational Ecology and Environmental Science Group, Computational Science Laboratory, Microsoft Research, Cambridge, United Kingdom; University of Kent, United Kingdom

## Abstract

Species distribution models (SDMs) trained on presence-only data are frequently used in ecological research and conservation planning. However, users of SDM software are faced with a variety of options, and it is not always obvious how selecting one option over another will affect model performance. Working with MaxEnt software and with tree fern presence data from New Zealand, we assessed whether (a) choosing to correct for geographical sampling bias and (b) using complex environmental response curves have strong effects on goodness of fit. SDMs were trained on tree fern data, obtained from an online biodiversity data portal, with two sources that differed in size and geographical sampling bias: a small, widely-distributed set of herbarium specimens and a large, spatially clustered set of ecological survey records. We attempted to correct for geographical sampling bias by incorporating sampling bias grids in the SDMs, created from all georeferenced vascular plants in the datasets, and explored model complexity issues by fitting a wide variety of environmental response curves (known as “feature types” in MaxEnt). In each case, goodness of fit was assessed by comparing predicted range maps with tree fern presences and absences using an independent national dataset to validate the SDMs. We found that correcting for geographical sampling bias led to major improvements in goodness of fit, but did not entirely resolve the problem: predictions made with clustered ecological data were inferior to those made with the herbarium dataset, even after sampling bias correction. We also found that the choice of feature type had negligible effects on predictive performance, indicating that simple feature types may be sufficient once sampling bias is accounted for. Our study emphasizes the importance of reducing geographical sampling bias, where possible, in datasets used to train SDMs, and the effectiveness and essentialness of sampling bias correction within MaxEnt.

## Introduction

Species distribution models (SDMs), which predict a species’ probability of occurrence across a landscape by relating documented locations of that species to environmental information, are frequently used in ecological, environmental and climate change research [1], [2], [3], [4], [5]. Species location data are increasingly made available by museums, herbaria, and other scientific institutions [6], [7], [8] via open-access data portals such as the Global Biodiversity Information Facility (GBIF; www.gbif.org), providing a wealth of information about the known presences of organisms (but not about known absences). There is also a ready supply of environmental information, including global databases of climate and digital elevation models [9] and user-friendly software packages. These technological advances mean that, as never before, SDMs are being used in ecological research and conservation planning. However, users of SDM software are faced with a variety of options, and it is not always obvious whether selecting one option over another has a major effect on model performance. This paper explores the consequences of correcting for geographical sampling bias and non-automatically selecting model functional forms on the predictive ability of MaxEnt, one of the best performing species distribution modelling techniques for analysis of presence-only data [10], [11], [12], [13].

Accounting for the effects of geographical sampling bias in the acquisition of data can be critical to the accuracy of SDMs generated from presence-only datasets [14], but options to correct for sampling bias are not always applied [15]. Samples are often collected from relatively accessible locations near roads, urban settlements and rivers, rather than systematically or randomly, so their sampled localities may not be representative of the true range of environmental conditions in which the species occurs [16], [17]. Such geographical sampling bias is a characteristic of most specimen locality data available from open access data portals [18]. Failure to correct for geographical sampling bias can result in a SDM that reflects sampling effort rather than the true distribution of a species [14]. Species distribution modellers aiming to use data from open access data portals are therefore confronted with the challenge of correcting for the likely influence of geographical sampling bias. Phillips et al. [14] recently developed a method for dealing with geographical sampling bias when modelling species distributions with presence-only data. Their approach is to generate background data (sometimes referred to as “pseudo-absences”) which has a similar geographical sampling bias to that of the presence data (the background data is the set of geographical locations that will be used to train the SDM). This is achieved by creating a sampling bias grid representing relative survey effort across the landscape, using the presence localities of a broader group of species within the region of interest (e.g. all bird species if modeling a single bird species), which is used in the SDM training algorithm [14]. Using independent presence-absence data to evaluate their models, they demonstrated that predictive accuracy improved when using this approach. Although not focussing on correcting for sampling bias, other studies have also explored different strategies for controlling the selection of background data to improve SDM performance [19], [20], [21], [22], [23].

The default option of MaxEnt allows the software to automatically select functional forms to describe species’ responses to environmental conditions, but users can select from a list of functional forms (i.e. linear, quadratic, threshold, hinge, product, and categorical) and allow different functional forms for different environmental variables. Although MaxEnt is designed to balance model goodness-of-fit against complexity [24], it is often the case that a variety of functional forms are combined to build a model, resulting in complex environmental response functions that are difficult to interpret from an ecological perspective. To the best of our knowledge, no study has compared the relative predictive performances of MaxEnt models that have been deliberately restricted to a distinct subset of the possible functional forms (resulting in simpler and more interpretable models) and evaluated using independent data on real species. This has particular relevance when fitting models to species with different amounts of occurrence data because it is unclear how much model performance is improved by allowing for more complex environmental response functions. Warren and Seifert [25] showed for model-generated data that overly complex or simple models fitted by MaxEnt had relatively poor predictive performance for a range of metrics. However, in their study model complexity was varied by adjusting a regularisation (*β*) parameter. This makes the influence of directly restricting the number of different types of functional forms unclear because adjusting this parameter can restrict both the number and type of functional forms included in the model. Given that the type of functional forms included is influenced by the quantity of data in MaxEnt, we wanted to see if we gained improved predictive performance when more abundant data allowed us to fit more complex models compared to simpler models.

This study explores the consequences of correcting for geographical sampling bias, and selecting specific functional forms, on the predictive performance of models generated by MaxEnt. We model the distribution of New Zealand tree ferns using presence-only data from two sources: a small dataset from herbarium collections and a much larger dataset from ecological surveys. These data were collected in different ways and are inherently different in their spatial properties and geographical biases. An advantage of working with New Zealand tree ferns is that we had access to an independent presence-absence dataset with which to evaluate predictive performance of our models; importantly, this independent dataset was collected from permanent plots at the intersections of a grid encompassing all of New Zealand’s indigenous forests and shrublands, so provided us a geographically unbiased sample with which to evaluate model performance. Our analyses indicate that predictive performance is greatly improved by correcting for sampling bias, and that choosing simple functional forms can lead to similar performance as allowing the software to automatically select more complex ones.

## Materials and Methods

Distribution models were generated using MaxEnt (Version 3.3.2), which uses the principle of maximum entropy to discriminate the range of environments associated with species’ presences from the range of environments across the rest of the landscape [24], [26], [27]. The software requires the following data as inputs: geographical locations of species occurrences, gridded data of environmental variables and (optionally) a sampling bias grid. In our implementation we used MaxEnt to fit (“train”) a species distribution model to a random sample of 75% of the species occurrence data, with the remaining 25% of the data used to assess (“test”) model performance [28], [29]. By repeating this training and testing process on multiple (i.e. 40 model runs) random subsamples we used MaxEnt to assess uncertainty of the SDM predictions. In this study we also used data from an entirely different source to evaluate model performance. This is an important extra step, because our additional dataset is geographically and environmentally comprehensive, thus providing a more reliable evaluation of performance than obtained from subsampled data.

### Occurrence Data

The tree fern clade, comprised of 7 families and about 600 species, is distributed throughout wet tropical, subtropical and temperate regions that rarely freeze [30], [31]. We focussed on modelling the distribution of New Zealand tree ferns collectively, rather than working on the distributions of the country’s nine species [32]. Tree ferns produce vast quantities of tiny spores, so environmental constraints rather than dispersal limitation are likely to control distribution. Furthermore, New Zealand has dramatic topography with large climatic gradients, making it well suited for predicting species distributions as a function of environmental variables [20].

**Figure 1:**
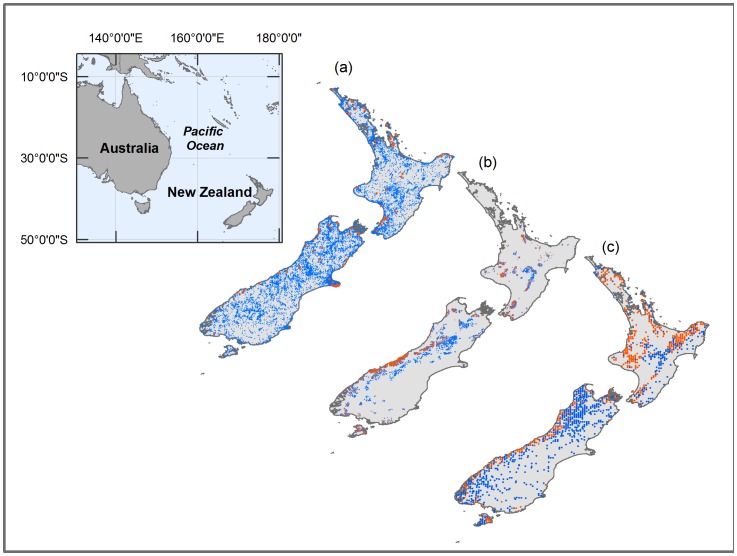
Data locations in New Zealand. Tree fern occurrence locations (orange) and “absence” locations (blue) are based on a) herbarium data extracted from GBIF; b) NVS ecological survey data extracted from GBIF, and c) LUCAS plot data. In the case of the herbarium and NVS datasets, “absences” are background locations based on locations of other vascular plants; in the case of the LUCAS dataset, true absences are shown.

Georeferenced plant occurrence data were accessed from (a) herbarium collections and (b) the National Vegetation Survey databank (NVS) via the GBIF portal. The herbarium collections are mostly from the New Zealand National Plant Herbarium, sampling much of the New Zealand landmass, including grasslands and agricultural land in dry eastern regions. NVS serves as New Zealand’s main repository for ecological data, containing surveys conducted over a period of 50 years [33]; the databank contains presence and absence locations but GBIF only provides information on the occurrence locations. In total, the herbarium dataset had 86 tree fern records and the NVS dataset had 5,874 records in which only one occurrence was used per pixel (∼ 1 km; Figure 1a and 1b).

An independent dataset containing tree fern presence and absence locations was used to evaluate the accuracy of the MaxEnt models trained on the herbarium and NVS datasets. We extracted tree fern presence (474) and absence (760) locations (Figure 1c) from the New Zealand Land Use and Carbon Analysis System (LUCAS) dataset for natural forests (http://www.mfe.govt.nz/issues/climate/lucas/). Forests were sampled nationally for the purpose of estimating carbon storage in New Zealand’s indigenous forests and shrublands, but this dataset also provides an overview of vascular plant diversity of these vegetation types [34], [35], [36]. Sampling plots were established on an 8×8 km grid spanning the entire country. At every forested intersection on the grid, a 20×20 m plot was established and the presence/absence of plant species recorded [37], resulting in approximately 1300 permanent plots. The LUCAS dataset was completely independent of the data used to train the model and was used only for model evaluation.

### Environmental Variables

We obtained mean annual bioclimatic variables from the Worldclim database, version 1.4 **(** http://www.worldclim.org; [38]). We supplemented these climate variables with three water balance variables: mean annual potential evapotranspiration (PET; Trabucco & Zomer [39]), actual evapotranspiration (AET; Trabucco & Zomer [40]), and annual water deficit calculated as PET- AET [41]. We added the water balance variables because of research indicating their importance in constraining plant distributions [41], [42]; data layers were extracted from the CGIAR consortium for spatial information (http://www.cgiar-csi.org/). All variables had a 30 arc second (∼1 km at the Equator) spatial resolution.

In all predictive modelling it is prudent to select explanatory variables that are not closely correlated [43]. A combination of expert knowledge, previous studies of fern diversity across New Zealand [44], and guidance from statistical analysis (correlation, hierarchical clustering, and principal components analyses) were used for variable selection. We included only variables with a Pearson correlation coefficient of less than 0.85. The variables selected for modelling were annual precipitation, minimum temperature, water deficit, AET, and temperature seasonality. Annual precipitation has long been recognized as a major determinant of species’ distributions [45]. Water deficit and minimum temperature represent the species’ tolerance to drought and cold temperatures. AET is the amount of water loss given existing evaporative energy in a system and the available water provided by precipitation and storage in the soil [46]. Temperature seasonality potentially differentiates sites with similar mean temperature, thus representing species’ sensitivity to temperature fluctuations.

### Sampling Bias Grids

We produced separate sampling bias grids for the herbarium and NVS datasets. In both cases, we totalled the number of vascular plant records found within grid cells which had the same resolution and positioning as the environmental grid cells (i.e. 30 arc seconds). Pixels without any plant occurrences were designated as having “no data” and were automatically excluded by MaxEnt.

### Model-fitting

The benefits of correcting for geographical sampling bias were evaluated by comparing SDMs that were fitted with and without the use of a sampling bias grid. Models fitted without the grid used “background data” comprised of random locations from anywhere in New Zealand, whereas models which corrected for sampling bias used “background data” comprised of random locations weighted by the sampling bias grid, as recommended by Phillips et al. [14].

MaxEnt provides the option to use a range of functional forms to describe the relationship between logit (probability of occurrence) and an environmental variable. These functional forms (known as “feature types”) are: Linear (L), Quadratic (Q), Hinge (H), Product (P), Threshold (T) and categorical (see [26], [27] for detailed descriptions). Furthermore, MaxEnt provides the option to allow different feature types to be used for different environmental variables. The default setting is “Auto feature”, which uses an algorithm to determine the most suitable complexity based on the number of presence records used for model training; at least 80 presence records are required in the training data to justify including all feature types under the “Auto” setting. We investigated the extent to which permitting MaxEnt to use different feature types influenced the predictive accuracy of the resulting models. The feature type combinations investigated were L, Q, H, T, LQ, HQ, LQP, LQT, QHP, QHT, QHPT, and Auto.

### Measures of Model Performance

The discrimination ability of an SDM is its ability to correctly distinguish between sites associated with presences and absences (or the background in the case of MaxEnt). We used the frequently applied Area Under the Curve (AUC) metric to evaluate SDM discrimination ability [9], [28]; an AUC value of 1.0 is considered a perfect prediction and a value of 0.5 or less is consider a prediction no better than random. We compared internal MaxEnt AUC calculations from the randomly withheld test data and AUC calculations from the independent LUCAS data. In addition, we used the Pearson correlation coefficient (COR: in this context is referred to as point biserial correlation; [10], [47]) to quantify the degree of correlation between model-predicted probabilities of occurrence and presences and absences of the LUCAS data. COR was calculated using R 2.10.1 [48] and the AUC was calculated from using the ROCR package [49].

Another aspect of evaluating model performance is to quantify how observed prevalence over a landscape varies with the predicted probability of occurrence [50]. For example, if we have a true occurrence probability of 0.2 then we should expect only 20% of such sites to contain presence observations. The question is how prevalence scales with the probability of occurrence predicted by the SDMs produced by MaxEnt; for example, do sites with occurrence probability of 0.4 really contain twice as many presences as those with probability of 0.2? Presence only calibration (POC) plots characterise how the actual prevalence scales with occurrence probability and Phillips and Elith [51] have demonstrated the usefulness of POC plots in which background locations are treated similarly to absences. Whilst the probability predicted by a presence-only SDM cannot be used alone to predict the true prevalence [51], the POC plot can be used to indicate the extent to which the predicted probability of presence scales with the actual prevalence of presence points in the background data.

As a final test of model performance we assessed the spatial patterns of predicted presences and absences of the LQ models using the independent LUCAS data. Implementing this requires choosing a probability threshold at which values above the threshold are predicted as a presence and values below as an absence. The threshold selection method we chose was “maximum (sensitivity+specificity)” [9], which gives the highest total value of sensitivity (proportion of actual presences that are accurately predicted) and specificity (proportion of actual absences that are accurately predicted).

## Results

### Correcting for Geographical Sampling Bias

The predictive performance of LQ models was greatly improved by correcting for geographical sampling bias using the methods of Phillips et al. [14]. Correcting bias in the herbarium and NVS datasets led to dramatic increases in AUC and COR values when model predictions were compared with observed tree fern presences and absences in the independent LUCAS dataset (Table 1). Correcting for geographical sampling bias approximately halved the false-absence and false-presence error rates of distribution maps predicted with the NVS dataset (Table 2; Figure S1), and approximately halved the false absence rate of distribution maps predicted with the herbarium dataset, although paradoxically the false presence rate increased following the correction (Table 2). The percentage of New Zealand predicted to be occupied by tree ferns was similar for the two datasets when geographical sampling bias had been corrected, but differed substantially when no correction was applied (Table 2).

**Table 1:** Effects of correcting for geographical sampling bias on the predictive performance of New Zealand tree fern distribution models trained on herbarium and NVS datasets.

	Not correcting for sampling bias				Correcting for sampling bias			
	AUC		COR		AUC		COR	
Herbarium dataset	0.787	**±**0.012	0.474	**±**0.020	0.851	**±**0.004	0.588	**±**0.008
NVS dataset	0.587	**±**0.003	0.165	**±**0.005	0.837	**±**0.004	0.549	**±**0.005

MaxEnt was used to fit the models (feature type = LQ) and model performance indicated by mean (±1 standard deviation) AUC and COR values, evaluated by using the independent LUCAS dataset.

**Table 2:** Effects of correcting for geographical sampling bias on the rates of false presences and absences, and on the predicted extent of tree ferns (as a percentage of the total land area of New Zealand).

	Not correcting for sampling bias			Correcting for sampling bias		
	Falseabsences† (%)	False presences‡ (%)	Percentage of NZpredicted to beoccupied	Falseabsences (%)	Falsepresences (%)	Percentage of NZpredicted to beoccupied
Herbarium dataset	12.4	41.2	45.4	20.3	19.5	30.9
NVS dataset	19.8	64.1	34.5	12.2	30.0	35.9

Models were fitted to two datasets (herbarium and NVS) using MaxEnt with the feature type set as “LQ”. Model predictions were based on average predictions from the 40 runs and evaluated by using the LUCAS dataset.

† False presences occur when a model predicts a species as present whilst observed data indicate it is absent.

‡ False absences occur when a model predicts a species as absent whilst observed data indicate it is present.

**Figure 2:**
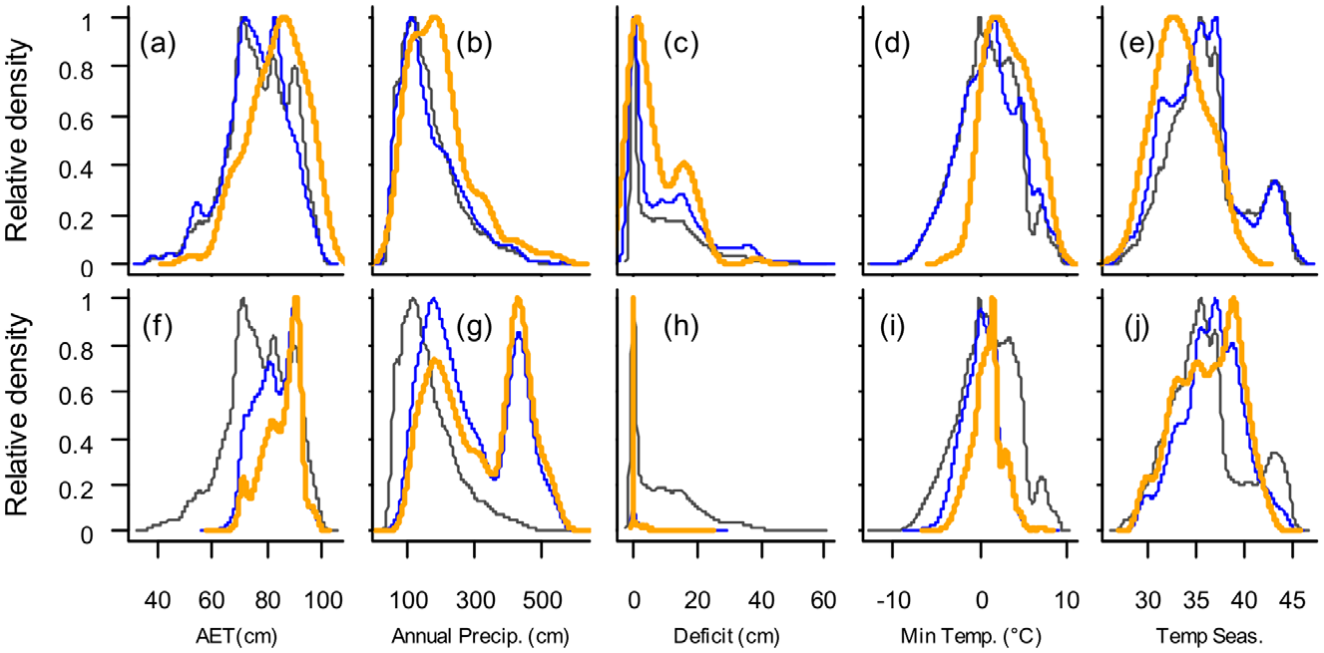
Density distribution plots of environmental variables. Tree fern occurrences (orange) and background locations are based on locations of other vascular plants (blue) compared to all NZ locations (∼1 km resolution; black) for the herbarium dataset (upper row) and the NVS dataset (lower row). Temperature seasonality is represented as standard deviations multiplied by 10.

**Figure 3:**
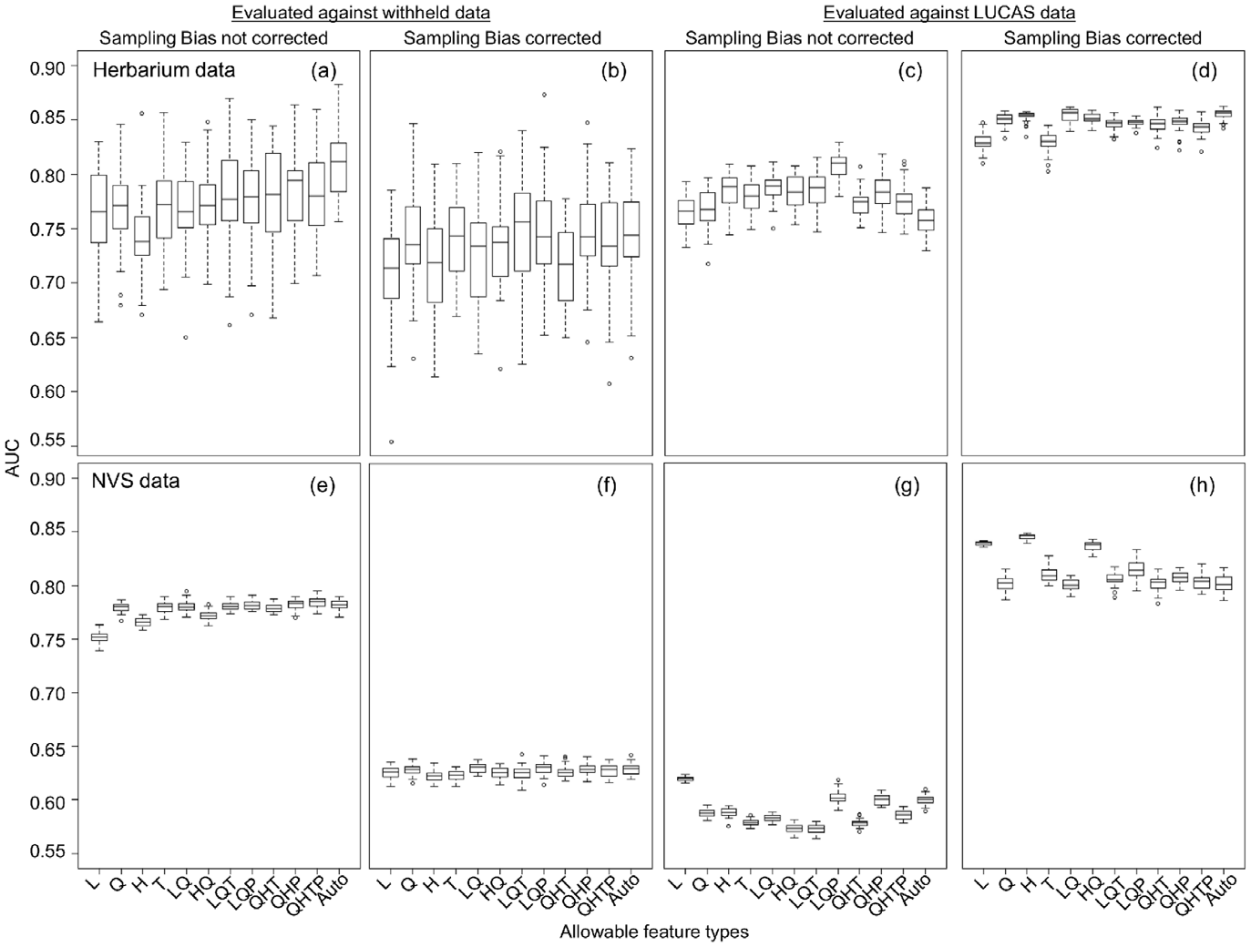
Comparison of presence-only calibration (POC) plots. MaxEnt LQ models were trained on (a) herbarium and (b) NVS data, correcting for geographical sampling bias; plots were derived from the average predictions of 40 runs. Values above the linear diagonal signify model underestimation of species prevalence and values below the line signifies overestimation of species prevalence. The calibration curve is shown in cyan and the orange lines represent ±2 standard deviations. Presence and background data are marked at the bottom of each graph at their corresponding predicted probabilities of presence: presences are orange and background data are black.

Performance improvements arising from sampling bias correction are particularly strong for the NVS dataset. In the case of the herbarium dataset, the frequency distributions of vascular plant records along five environmental axes were virtually identical to the frequency distributions of the New Zealand landmass along these axes (blue vs black lines in Figure 2a–e), indicating that the herbarium collections were broadly representative of the environmental conditions found in New Zealand. However, the equivalent frequency distributions for NVS were quite distinct from the frequency distributions of the New Zealand landmass (blue vs black lines in Figure 2f–j), indicating that surveys were biased towards wetter parts of the country and under-represented the coldest and warmest regions. We further assessed the performance of LQ models with sampling bias correction using POC plots (Figure 3). The relative probability of species presence data in the herbarium dataset tends to increase in direct proportion to the probability of presence predicted by LQ models, while the relative probability of species presence data in the NVS dataset indicates that the models tend to under-predict the relative prevalence of presences in the dataset at low predicted probabilities of presence, and then over predict at high probabilities of presence. Whilst there were benefits to correcting for sampling bias in both datasets, they were far greater for NVS because of the strong geographical bias within this dataset.

The frequency distributions of tree fern records along five environmental axes were quite distinct from those of other vascular plant records (orange vs blue lines in Figure 2), indicating that the ferns occupy only part of the available environmental niche space, although this distinction was clearest in the case of the herbarium dataset. It is well known that tree ferns grow in wet regions which do suffer from long periods of freezing in the winter months, and are particularly common in warmer regions of the southern hemisphere [44]. Natural history observation regarding tree ferns are borne out in Figure 2, and also in the environmental responses obtained by MaxEnt modelling (Figure S2 a and c): for both datasets, the probability of occurrence is seen to increase with temperature (for which AET is a proxy), precipitation, and minimum temperature (with a sharp increase at about 0°C). However, the two analyses gave diametrically opposite predictions with regard to water deficit and temperature seasonality (Figure S2 a and c).

### Effects of Using Withheld Test Data for Model Evaluation

**Figure 4:**
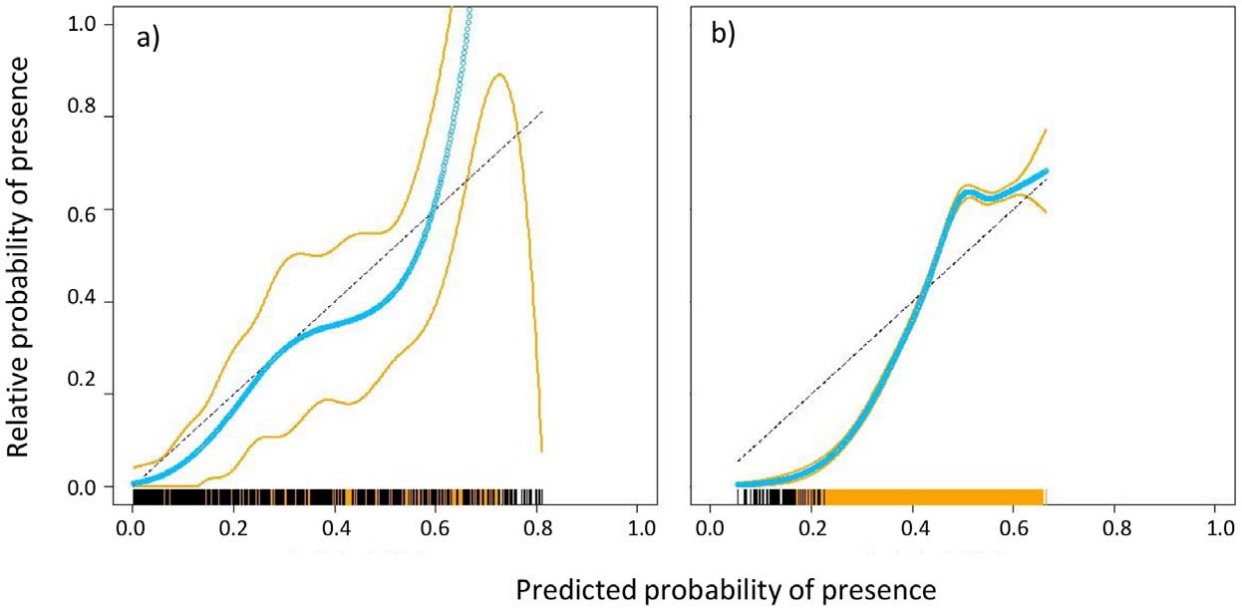
Box plots of AUC values. AUC values derived from MaxEnt models fitted using different functional forms (“feature types”) and two different training datasets: herbarium (a–d) and NVS (e–h). Evaluations are made using randomly withheld test data without and with correcting geographical sampling bias (a & e) and (b & f), respectively; evaluations are made using independent LUCAS data without and with correcting for sampling bias are (c & g) and (d & h), respectively. Box plots indicate variation in AUC among 40 runs (boxes encompass 25^th^ and 75^th^ percentiles, whiskers approximate 99% of the data range, points are outliers).

It is common practice to withhold 25% of occurrence data from the training process so that it can be used for testing model performance, but our results indicate that this procedure could be problematic if interpreted improperly. Correcting for geographic sampling bias led to decreases in AUC values when withheld data were used for testing model accuracy (Figure 4b, f) as well as a decrease in COR values (results not shown). Thus using withheld data to evaluate model performance with respect to sampling bias does not indicate the increase in SDM performance at predicting the true species distribution (see discussion for further explanation).

### Effects of Model Complexity

The choice of feature types had relatively minor effects on model predictive performance (Figure 4). Average AUC increased slightly when models were fitted with more complex feature types, when assessed against LUCAS data, although this effect was minor compared to correcting for sampling bias (Figure 4c, d, g, h). The auto feature models performed slightly better than the LQ models for the herbarium dataset (mean AUC = 0.855 vs 0.851 respectively; Welch t-test t_76_ = 3.60; *P*<0.001) but was considerably worse for the NVS dataset (mean AUC = 0.801 vs 0.837 respectively, t_61_ = 26.42, *P*<0.001). The environmental response curves predicted by simple LQ models were much smoother than those obtained from auto feature models (Figure S2), suggesting that the auto feature option may have picked up local idiosyncratic effects rather than broad physiological responses.

### Spatial Patterns of Errors in Model Predictions

**Figure 5:**
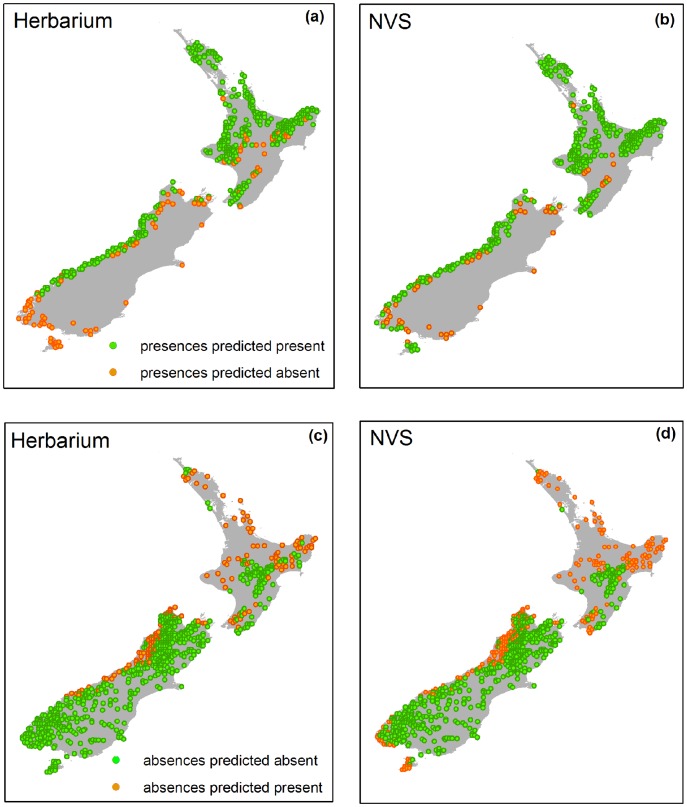
LUCAS presence/absence locations with predicted presences and absences generated from average LQ model predictions (with geographical sampling bias correction). Correct agreement between predicted presences/absences and LUCAS presences/absences are shown in green and incorrect agreements are shown in orange. LUCAS presence locations are shown with predictions from (a) herbarium dataset and (b) NVS dataset, LUCAS absence locations are shown with predictions from (c) herbarium dataset and (d) NVS dataset.

Distinct spatial patterns of false presences and false absences are apparent in LQ models fitted to the NVS and herbarium datasets, applying the sampling bias correction. Visual inspection of models fitted to the herbarium dataset indicated low predictive performance in the far southern region of the South Island and Stewart Island – there was a very high rate of false absence (100%; Figure 5a), with almost half of all false absences coming from this region. In contrast the NVS dataset had a higher false presence rate on the North Island (57.5%; Figure 5d) than the herbarium dataset (42.5%; Figure 5c).

## Discussion

### Correcting for Geographical Sampling Bias

Our analyses show clearly that the method proposed by Phillips et al. [14] to correct for sampling bias greatly improves the predictive performance of SDMs. This was true for the strongly biased NVS dataset, and also for the more subtly biased herbarium collections. A number of other studies have shown the importance of the background data selection method through a variety of SDM techniques [19], [20], [21], [22], [23], [52] and studies have begun to also evaluate MaxEnt [12], [14], [53], [54], [55]. Our study supports the use of the correction method devised by Phillips et al. [14], which is available as an option in the MaxEnt software.

The benefits of correcting for sampling bias were only apparent when we assessed model performance using independent presence-absence data (Table 1 and Figure 4). Paradoxically, when 25% of the training dataset is withheld for model evaluation, predictive performance appeared to decrease when sampling bias corrections were introduced (Figure 4). This is because the training and test datasets possess the same geographical sampling bias. It may be that environmental response curves obtained without sampling bias correction may partly reflect underlying variation in sampling intensity rather than pure environmental responses, but the models appear to perform well when evaluated with withheld training data, because the test and training datasets are similarly biased [14]. Conversely, model performance appears to fall when the correction is applied, because most realistic environmental response curves are obtained, and these may not predict presences in densely sampled regions as a matter of course [14]. Clearly then, performance statistics derived from withheld data cannot be used to justify whether or not to correct for bias. We suggest that geographic bias correction should become the default option and that researchers should provide a strong justification if they choose to avoid it.

Our investigation supports the suggestion that the completeness of sampling with respect to the environmental gradients is more important for performance than sample size [56]. Models trained on the small herbarium dataset outperformed those trained on the large-but-biased NVS dataset, even after correcting for geographical bias: AUC and COR values were higher for the herbarium models, POC curves departed little from the expected line, and errors rates in the North Island were lower (Figure 5). The environmental response functions of models fitted using the herbarium dataset and restricting MaxEnt to linear and quadratic features also support our ecological understanding of the abiotic determinants of tree fern distributions (Figure S2). The relatively poorer performance of models trained on the NVS data is likely related to using occurrence data from a restricted range of environmental space. This is consistent with other studies that have shown that model performance is influenced by the relationship between the occurrence data environmental range and the background data (pseudo-absence) environmental range [52], [57].

Our analyses of the spatial variation in the predictive performance indicate clear differences between datasets, highlighting the value of this approach [58]. The performance of the models trained on the herbarium data is poor in the very south of New Zealand because this region was poorly represented, with only a single collection out of 80 in the dataset. There is more variation in the AET response curves at low temperatures (i.e. at low AET, Figure S2), also reflecting this lack of data. This underlines that the predictive ability of SDMs can be severely limited by the lack of representative occurrence data from the range of environments in which the species occurs and emphasises why caution should be placed in SDM predictions that are at the edge or extrapolated beyond the range of environments that are associated with the training occurrence data [57].

### Model Complexity

Increasing the allowable complexity of the functional forms had little effect on model performance, implying that MaxEnt might be able to produce models with similar levels of performance with combinations of much simpler feature types (linear and quadratic in our case) than obtained using the default MaxEnt settings. Simple functional forms are easier to interpret from an ecological perspective and more readily translated into other modelling paradigms, such as process-based models, and prevent locally idiosyncratic responses which may be specific to the particular datasets rather than reflecting general features of an organism’s physiological responses to the environment. However, Warren and Seifert [25] recently demonstrated that over-parameterization was typically less important than under-parameterization for model performance using simulated datasets. We suggest that MaxEnt users fit models with the default auto feature option but also try fitting simple functional forms (e.g. LQ); if the simpler functions perform as well as the automatic option and are more easily interpreted, it may be preferable to report these results.

### Conclusions

Our findings strongly endorse the method of Phillips et al [14] for correcting sampling bias in presence-only species distribution models. Even so, our bias-corrected models had false presence and absence rates of around 20%, and these error rates were greatest in particular regions of New Zealand, so care is needed when applying this approach for species conservation. A novel implication in our study for those training SDMs on data from online data portals such as GBIF is that the appropriate sampling bias grid may differ depending on the data provenance (NVS versus herbarium data in our case). We found that the smaller herbarium dataset produced more accurate distribution models than the much larger ecological survey data, because of the strong spatial biases in the NVS dataset. Thus, we encourage a careful evaluation of the potential geographical sampling bias before building a SDM. Additionally, we find that the use of simpler feature types may result in models with similar levels of predictive performance to those in which a wider range of feature types were possible. These simpler models have the added benefit of being easier to interpret and thereby aid the understanding of a species’ ecology, which may be particularly valuable in the case of the many potentially threatened plant species for which ecological information is lacking [59], [60].

## Supporting Information

Figure S1 **Average MaxEnt predictions from 40 runs of LQ models built using the herbarium and NVS datasets when geographical sampling bias is and is not corrected.** Using the MaxEnt logistic output, blue colours indicate a higher “probability of occurrence” (suitability) while the orange colours indicate lower probabilities.
(TIF)

Figure S2 **Response plots showing the relationship between predicted probability of presence and environmental variables when all other variables are held at their empirical averages.** Models were fitted using LQ features trained on herbarium data (a) and NVS data (c), and models were fitted using Auto features trained on herbarium data (b) and NVS data (d). Geographical sampling bias was corrected in all cases. The response curve is shown in black and the grey areas represent 95% confidence intervals from 40 replicated runs.
(TIFF)

## References

[1] Thuiller W, Albert C, Araujo MB, Berry PM, Cabeza M, et al (2008) Predicting global change impacts on plant species’ distributions: Future challenges. Perspectives in Plant Ecology Evolution and Systematics 9: 137–152.

[2] Elith J, Leathwick JR (2009) Species Distribution Models: Ecological Explanation and Prediction Across Space and Time. Annual Review of Ecology Evolution and Systematics 40: 677–697.

[3] Guisan A, Thuiller W (2005) Predicting species distribution: offering more than simple habitat models. Ecology Letters 8: 993–1009. 10.1111/j.1461-0248.2005.00792.x 34517687

[4] Araújo MB, Luoto M (2007) The importance of biotic interactions for modelling species distributions under climate change. Global Ecology and Biogeography 16: 743–753.

[5] Yates CJ, McNeill A, Elith J, Midgley GF (2010) Assessing the impacts of climate change and land transformation on Banksia in the South West Australian Floristic Region. Diversity and Distributions 16: 187–201.

[6] Graham CH, Ferrier S, Huettman F, Moritz C, Peterson AT (2004) New developments in museum-based informatics and applications in biodiversity analysis. Trends in Ecology & Evolution 19: 497–503. 16701313 10.1016/j.tree.2004.07.006

[7] Wisz MS, Hijmans RJ, Li J, Peterson AT, Graham CH, et al (2008) Effects of sample size on the performance of species distribution models. Diversity and Distributions 14: 763–773.

[8] Newbold T (2010) Applications and limitations of museum data for conservation and ecology, with particular attention to species distribution models. Progress in Physical Geography 34: 3–22.

[9] Franklin J (2009) Mapping Species Distributions: Spatial Inference and Prediction. Cambridge, UK: Cambridge University Press. 338 p.

[10] Elith J, Graham CH, Anderson RP, Dudik M, Ferrier S, et al (2006) Novel methods improve prediction of species’ distributions from occurrence data. Ecography 29: 129–151.

[11] Williams JN, Seo CW, Thorne J, Nelson JK, Erwin S, et al (2009) Using species distribution models to predict new occurrences for rare plants. Diversity and Distributions 15: 565–576.

[12] Mateo RG, Croat TB, Felicísimo ÁM, Muñoz J (2010) Profile or group discriminative techniques? Generating reliable species distribution models using pseudo-absences and target-group absences from natural history collections. Diversity and Distributions 16: 84–94.

[13] Hernandez PA, Graham CH, Master LL, Albert DL (2006) The effect of sample size and species characteristics on performance of different species distribution modeling methods. Ecography 29: 773–785.

[14] Phillips SJ, Dudik M, Elith J, Graham CH, Lehmann A, et al (2009) Sample selection bias and presence-only distribution models: implications for background and pseudo-absence data. Ecological Applications 19: 181–197. 19323182 10.1890/07-2153.1

[15] Yackulic CB, Chandler R, Zipkin EF, Royle JA, Nichols JD, et al.. (2012) Presence-only modelling using MAXENT: when can we trust the inferences? Methods in Ecology and Evolution: early view.

[16] Reddy S, Davalos LM (2003) Geographical sampling bias and its implications for conservation priorities in Africa. Journal of Biogeography 30: 1719–1727.

[17] Kadmon R, Farber O, Danin A (2004) Effect of roadside bias on the accuracy of predictive maps produced by bioclimatic models. Ecological Applications 14: 401–413.

[18] Hortal J, Jimenez-Valverde A, Gomez JF, Lobo JM, Baselga A (2008) Historical bias in biodiversity inventories affects the observed environmental niche of the species. Oikos 117: 847–858.

[19] Barbet-Massin M, Jiguet F, Albert CH, Thuiller W (2012) Selecting pseudo-absences for species distribution models: how, where and how many? Methods in Ecology and Evolution 3: 327–338.

[20] Zaniewski AE, Lehmann A, Overton JM (2002) Predicting species spatial distributions using presence-only data: a case study of native New Zealand ferns. Ecological Modelling 157: 261–280.

[21] Lutolf M, Kienast F, Guisan A (2006) The ghost of past species occurrence: improving species distribution models for presence-only data. Journal of Applied Ecology 43: 802–815.

[22] Engler R, Guisan A, Rechsteiner L (2004) An improved approach for predicting the distribution of rare and endangered species from occurrence and pseudo-absence data. Journal of Applied Ecology 41: 263–274.

[23] Chefaoui RM, Lobo JM (2008) Assessing the effects of pseudo-absences on predictive distribution model performance. Ecological Modelling 210: 478–486.

[24] Phillips SJ, Dudik M (2008) Modeling of species distributions with Maxent: new extensions and a comprehensive evaluation. Ecography 31: 161–175.

[25] Warren DL, Seifert SN (2011) Ecological niche modeling in Maxent: the importance of model complexity and the performance of model selection criteria. Ecological Applications 21: 335–342. 21563566 10.1890/10-1171.1

[26] Phillips SJ, Anderson RP, Schapire RE (2006) Maximum entropy modeling of species geographic distributions. Ecological Modelling 190: 231–259.

[27] Elith J, Phillips SJ, Hastie T, Dudik M, Chee YE, et al (2011) A statistical explanation of MaxEnt for ecologists. Diversity and Distributions 17: 43–57.

[28] Fielding AH, Bell JF (1997) A review of methods for the assessment of prediction errors in conservation presence/absence models. Environmental Conservation 24: 38–49.

[29] Guisan A, Zimmermann NE (2000) Predictive habitat distribution models in ecology. Ecological Modelling 135: 147–186.

[30] Bystriakova N (2008) *The Ecology and Biogeography of Tree Ferns* [PhD]. Cambridge, UK: University of Cambridge. 209 p.

[31] Korall P, Pryer KM, Metzgar JS, Schneider H, Conant DS (2006) Tree ferns: Monophyletic groups and their relationships as revealed by four protein-coding plastid loci. Molecular Phylogenetics and Evolution 39: 830–845. 16481203 10.1016/j.ympev.2006.01.001

[32] Korall P, Conant DS, Metzgar JS, Schneider H, Pryer KM (2007) A molecular phylogeny of scaly tree ferns (Cyatheaceae). American Journal of Botany 94: 873–886. 21636456 10.3732/ajb.94.5.873

[33] Wiser SK, Bellingham PJ, Burrows LE (2001) Managing biodiversity information: development of New Zealand’s National Vegetation Survey databank. New Zealand Journal of Ecology 25: 1–17.

[34] Coomes DA, Allen RB, Scott NA, Goulding C, Beets P (2002) Designing systems to monitor carbon stocks in forests and shrublands. Forest Ecology and Management 164: 89–108.

[35] Richardson SJ, Peltzer DA, Hurst JM, Allen RB, Bellingham PJ, et al (2009) Deadwood in New Zealand’s indigenous forests. Forest Ecology and Management 258: 2456–2466.

[36] Wiser SK, Hurst JM, Wright EF, Allen RB (2011) New Zealand’s forest and shrubland communities: a quantitative classification based on a nationally representative plot network. Applied Vegetation Science 14: 506–523.

[37] Payton IJ, Newell CL, Beets PN (2004) New Zealand Carbon Monitoring System: indigenous forest and shrubland data collection manual. Manaaki Whenua – Landcare Research and Forest Research, NZ.

[38] Hijmans RJ, Cameron SE, Parra JL, Jones PG, Jarvis A (2005) Very high resolution interpolated climate surfaces for global land areas. International Journal of Climatology 25: 1965–1978.

[39] Trabucco A, Zomer RJ (2009) Global Aridity Index (Global-Aridity) and Global Potential Evapo-Transpiration (Global-PET) Dataset. CGIAR-CSI GeoPortal, Available: http://www.csi.cgiar.org. Accessed: 2012 Dec 19.

[40] Trabucco A, Zomer RJ (2010) Global High-Resolution Soil-Water Balance Geospatial Database. CGIAR-CSI GeoPortal Available: http://www.cgiar.csi.org. Accessed December 19 2012.

[41] Stephenson NL (1998) Actual evapotranspiration and deficit: biologically meaningful correlates of vegetation distribution across spatial scales. Journal of Biogeography 25: 855–870.

[42] Stephenson NL (2000) Climate, Vegetation, and Considerations for Restoration. U.S. Geological Survey Open-File Report 00–62.

[43] Zuur AF, Ieno EN, Elphick CS (2010) A protocol for data exploration to avoid common statistical problems. Methods in Ecology and Evolution 1: 3–14.

[44] Lehmann A, Leathwick JR, Overton JM (2002) Assessing New Zealand fern diversity from spatial predictions of species assemblages. Biodiversity and Conservation 11: 2217–2238.

[45] Woodward FI, Williams BG (1987) Climate and plant distribution at global and local scales. Plant Ecology 69: 189–197.

[46] Frank DA, Inouye RS (1994) Temporal Variation in Actual Evapotranspiration of Terrestrial Ecosystems - Patterns and Ecological Implications. Journal of Biogeography 21: 401–411.

[47] Elith J, Graham CH (2009) Do they? How do they? WHY do they differ? On finding reasons for differing performances of species distribution models. Ecography 32: 66–77.

[48] R Development Core Team (2010) *R: a Language and Environment for Statistical Computing* Vienna, Austria: R Foundation for Statistical Computing. Available: http://www.r-project.org/. Accessed September 18 2011.

[49] Sing T, Sander O, Beerenwinkel N, Lengauer T (2005) ROCR: visualizing classifier performance in R. Bioinformatics. 21: 3940–3941. 10.1093/bioinformatics/bti623 16096348

[50] Pearce J, Ferrier S (2000) Evaluating the predictive performance of habitat models developed using logistic regression. Ecological Modelling 133: 225–245.

[51] Phillips SJ, Elith J (2010) POC plots: calibrating species distribution models with presence-only data. Ecology 91: 2476–2484. 20836469 10.1890/09-0760.1

[52] Stokland JN, Halvorsen R, Stoa B (2011) Species distribution modelling-effect of design and sample size of pseudo-absence observations. Ecological Modelling 222: 1800–1809.

[53] Wisz M, Guisan A (2009) Do pseudo-absence selection strategies influence species distribution models and their predictions? An information-theoretic approach based on simulated data. BMC Ecology 9: 8. 19393082 10.1186/1472-6785-9-8 PMC2680809

[54] VanDerWal J, Shoo LP, Graham C, William SE (2009) Selecting pseudo-absence data for presence-only distribution modeling: How far should you stray from what you know? Ecological Modelling 220: 589–594.

[55] Elith J, Kearney M, Phillips S (2010) The art of modelling range-shifting species. Methods in Ecology and Evolution 1: 330–342.

[56] Newbold T, Reader T, Zalat S, El-Gabbas A, Gilbert F (2009) Effect of characteristics of butterfly species on the accuracy of distribution models in an arid environment. Biodiversity and Conservation 18: 3629–3641.

[57] Thuiller W, Brotons L, Araujo MB, Lavorel S (2004) Effects of restricting environmental range of data to project current and future species distributions. Ecography 27: 165–172.

[58] Hanspach J, Kühn I, Schweiger O, Pompe S, Klotz S (2011) Geographical patterns in prediction errors of species distribution models. Global Ecology and Biogeography 20: 779–788.

[59] Rivers MC, Taylor L, Brummitt NA, Meagher TR, Roberts DL, et al (2011) How many herbarium specimens are needed to detect threatened species? Biological Conservation 144: 2541–2547.

[60] Brummitt NA, Bachman SP, Moat J (2008) Applications of the IUCN Red List: towards a global barometer for plant diversity. Endangered Species Research 6: 127–135.

