# Correction: The Effects of Sampling Bias and Model Complexity on the Predictive Performance of MaxEnt Species Distribution Models

**DOI:** 10.1371/annotation/35be5dff-7709-4029-8cfa-f1357e5001f5

**Published:** 2013-07-03

**Authors:** Mindy M. Syfert, Matthew J. Smith, David A. Coomes

Figures 3 and 4 were incorrectly switched. The figure appearing as Figure 3 belongs with the title and legend of Figure 4, and the figure appearing as Figure 4 belongs with the title and legend of Figure 3. The titles and legends themselves are in correct order. 

