# Supplementary figures and images for: The Effects of Sampling Bias and Model Complexity on the Predictive Performance of MaxEnt Species Distribution Models
Source: PLoS One. 2013 Feb 14;8(2):e55158. doi: 10.1371/journal.pone.0055158 (PMC3573023; doi:10.1371/journal.pone.0055158)

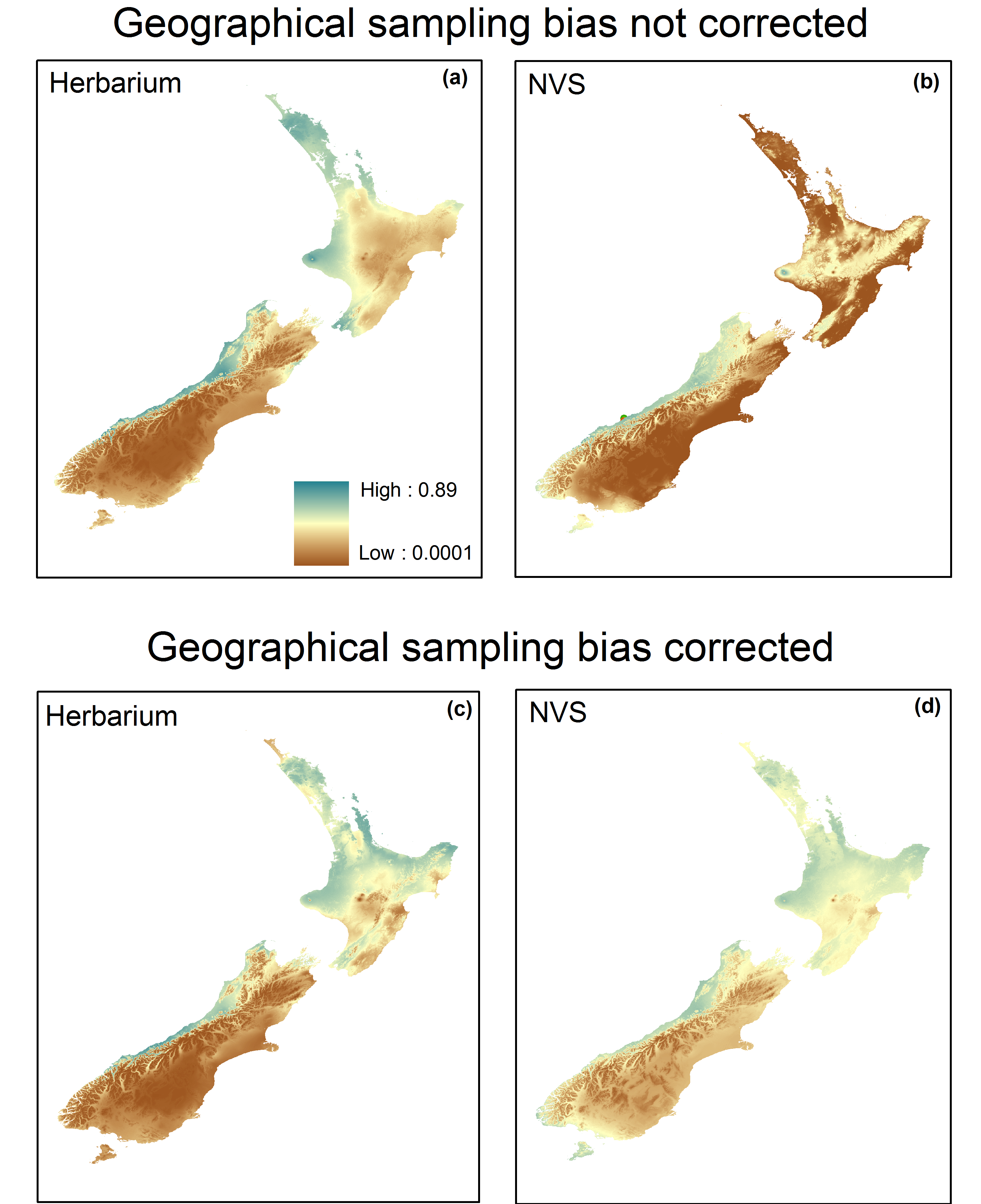

Supplement: Figure S1 — Average MaxEnt predictions from 40 runs of LQ models built using the herbarium and NVS datasets when geographical sampling bias is and is not corrected. Using the MaxEnt logistic output, blue colours indicate a higher “probability of occurrence” (suitability) while the orange colours indicate lower probabilities. (TIF) [file pone.0055158.s001.tif]

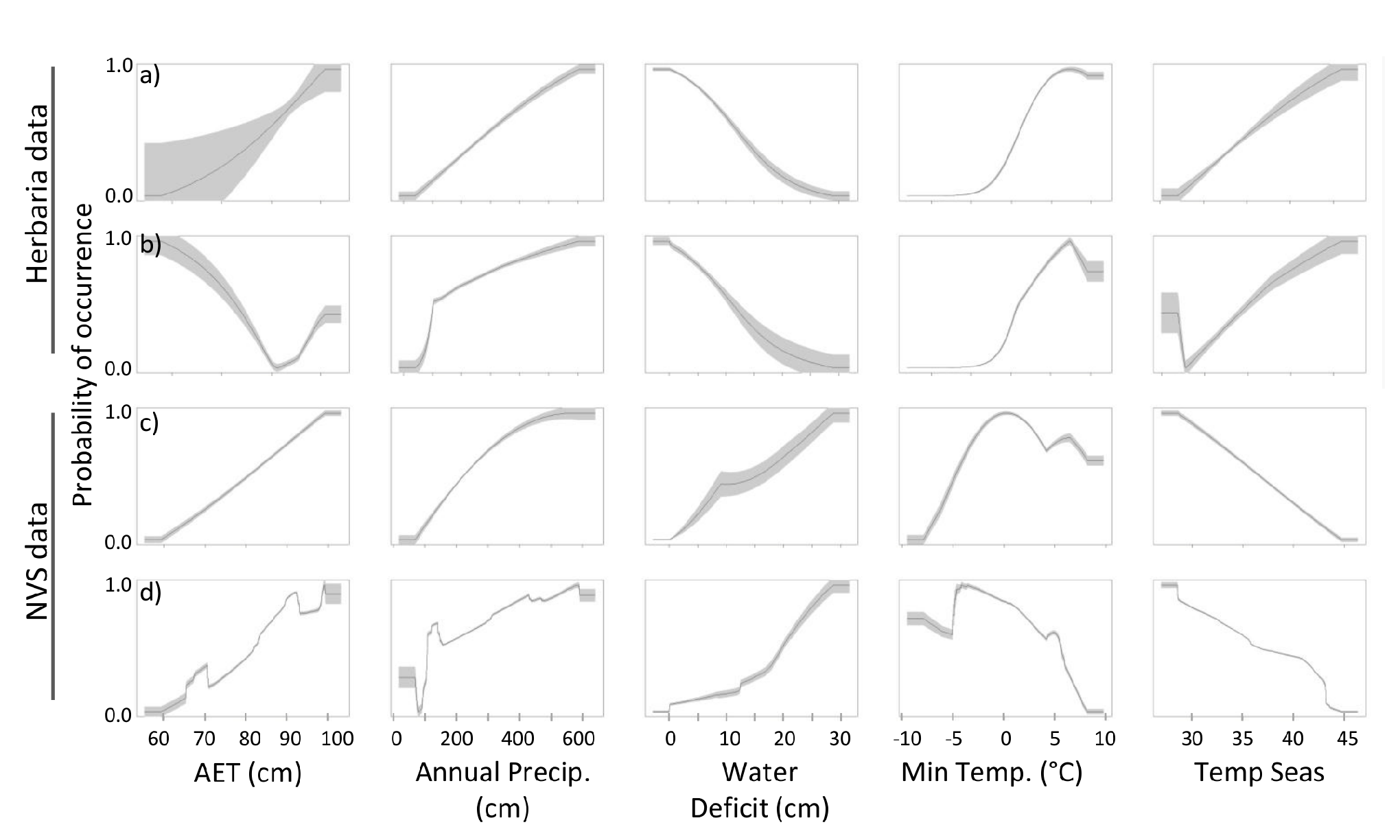

Supplement: Figure S2 — Response plots showing the relationship between predicted probability of presence and environmental variables when all other variables are held at their empirical averages. Models were fitted using LQ features trained on herbarium data (a) and NVS data (c), and models were fitted using Auto features trained on herbarium data (b) and NVS data (d). Geographical sampling bias was corrected in all cases. The response curve is shown in black and the grey areas represent 95% confidence intervals from 40 replicated runs. (TIFF) [file pone.0055158.s002.tiff]
